# Inkjet-Printed Electrode Enable Portable Electrochemical Immunosensing of Tau-441 for Early Alzheimer’s Screening

**DOI:** 10.3390/bios16020113

**Published:** 2026-02-10

**Authors:** Binglun Li, Chenghao Liu, Chenlu Gu, Shanshan Wei, Shiyong Li, Ziang Liu, Dongdong Zhao, Qunfeng Tang, Yun Chen, Zhencheng Chen

**Affiliations:** 1 School of Life and Environmental Sciences, Guilin University of Electronic Technology, Guilin 541004, China; 2 Guangxi Colleges and Universities Key Laboratory of Biomedical Sensors and Intelligent Instrument, Guilin 541004, China; 3 Guangxi Human Physiological Information Non-Invasive Detection Engineering Technology Research Center, Guilin 541004, China; 4 School of Electronic Engineering and Automation, Guilin University of Electronic Technology, Guilin 541004, China; 5 School of Artificial Intelligence Medicine, Guilin Medical University, Guilin 541199, China; 6 School of Physics and Technology, Guangxi Normal University, Guilin 541004, China

**Keywords:** Alzheimer’s disease, Tau-441, electrochemistry, inkjet-printed electrode, portable electrochemical platform

## Abstract

Early diagnosis of Alzheimer’s disease represents a critical clinical challenge, and the high-sensitive biomarkers measurement holds great potential for enabling early identification and intervention. This study proposes an electrochemical immunosensing strategy based on inkjet printing for the quantitative detection of Tau-441. Conductive patterns were formed by inkjet printing, followed by surface functionalization with gold nanoparticles to immobilize highly specific anti-Tau-441. This process created a stable and high affinity immunorecognition interface that enhances electron transfer and signal amplification. Furthermore, we developed and integrated a low-power portable detection platform to achieve a rapid detection process encompassing sample loading, signal acquisition, and on-device readout. The method shows a linear response from 50 fg/mL to 10 ng/mL and a limit of detection of 16 fg/mL (S/N = 3), with high specificity and good reproducibility. By combining scalable inkjet fabrication with a self-contained handheld reader, this method shortens the path from sample to result and offers a practical route for on-site screening and early intervention in Alzheimer’s disease.

## 1. Introduction

Alzheimer’s disease (AD) is a progressive neurodegenerative disorder and the most common cause of dementia worldwide [1,2,3,4]. Its typical pathological features are β-amyloid deposition and the neurofibrillary tangles caused by hyperphosphorylated Tau [5,6,7]. Abnormal Tau aggregation is closely associated with neuronal degeneration and cognitive impairment, and is a core pathological marker of AD [8,9]. Currently, diagnosis relies on imaging such as positron emission tomography, or on biomarker analysis in blood or cerebrospinal fluid [10,11,12,13,14,15]. However, these approaches require complex procedures, have long turnaround times and are costly, constraining large-scale clinical application. Therefore, sensitive and practical biomarker assays for early AD detection are needed to support early clinical screening [15,16,17].

Among various sensors, electrochemical biosensors have gradually become an ideal method for detecting early AD biomarkers due to their high sensitivity, low cost, and rapid response [18,19,20,21]. However, most studies still rely on screen-printed electrodes, whose fabrication often requires frequent screen changes and solvent-intensive cleaning. Moreover, the workflow commonly uses volatile, irritating, or toxic reagents, making routine operation and waste handling burdensome and user-unfriendly. In contrast, this study employs inkjet printing technology for electrode fabrication, which not only offers higher production efficiency and better pattern accuracy, but also is simple and flexible to operate [22,23].

The resulting electrochemical immunosensor couples nanomaterials with antibody recognition to achieve sensitive, rapid, and cost-effective quantification of low-abundance AD biomarkers, indicating strong potential for early diagnosis and broader deployment [24,25,26]. Gold nanoparticles (Au NPs) are used to modify the electrode because of their excellent conductivity and chemical stability, and antibodies serve as the recognition elements due to their high specificity and affinity [27,28,29,30,31,32,33]. Immunosensors are typically implemented in label-free or label-based formats, which differ in whether the antigen–antibody interaction is detected through intrinsic interfacial changes or through enzymatic and nanomaterial-assisted signal amplification. Building on recent advances in electrochemical platforms, which offer miniaturization and compatibility with printed architectures, our work employs a printed electrode with nanostructured Au to immobilize antibodies and enable selective Tau-441 detection [34].

In this study, we developed a portable electrochemical immunosensor for the rapid and sensitive quantitative detection of Tau-441 to support the early diagnosis of AD. It is mainly composed of three components:

(I) Inkjet-printed electrode (IPE): The surface of the working electrode (WE) was modified with Au NPs and immobilized with anti-Tau-441 to construct an efficient and sensitive recognition interface. Au NPs increased the attachment sites for antibodies and improved the detection sensitivity.
(II) Electrochemical sensing and acquisition module: This mainly includes a reference voltage circuit, a potentiostatic circuit, a weak current detection circuit and a filter circuit, which can quickly complete high-precision data acquisition and processing.
(III) Master control and communication module: The microcontroller unit (MCU) adopts the STM32F103C8T6 chip to complete the acquisition, processing and transmission of signals, and transmits Tau-441 information to the mobile terminal in real time.

The portable electrochemical immunosensor proposed in this study is suitable for rapid and sensitive quantitative detection of Tau-441. In actual serum sample tests, this sensor has demonstrated excellent detection performance and clinical applicability, providing a simple and efficient solution for the early large-scale screening of AD.

## 2. Experimental Section

All reagents and apparatus of the nanomaterials employed in this study can be found in the Supporting Information.

### 2.1. Preparation of the Immunosensor Electrode Printing

Electrode printing: The graphic design of the electrode was carried out using Adobe Illustrator 2024, and then inkjet-printing technology was adopted to achieve its large-scale production. Following the general method proposed by Boček et al., a planar graphene electrode substrate was fabricated using the HP P1106 printer through a layer-by-layer printing process. Subsequently, its performance was further optimized based on the film quality and conductivity [35]. Polyethylene glycol terephthalate (PET) was selected as the substrate material. Graphene with 35%wt Prussian blue (PB) was used as the working electrode ink. Ag/AgCl paste was printed to form the wires and the reference electrode, while graphene ink served as the counter electrode.

Electrode activation: The prepared IPE was immersed in 5 mL of 0.5 M H_2_SO_4_ solution and activated by electrochemical cyclic scanning at a scan rate of 100 mV/s, with a potential range of 0.2 V to 1.0 V, for 20 cycles.

**Figure 1:**
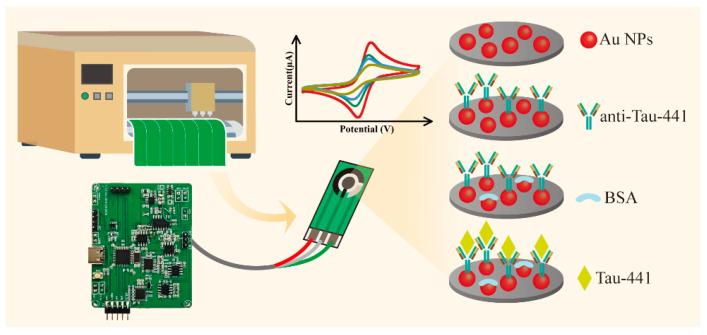
The preparation process of the electrochemical immunosensor based on anti-Tau-411/Au NPs.

Preparation of the IPE biosensor (Figure 1): First, 50 μL of a 0.5 mM HAuCl_4_ solution was dispensed onto the WE surface of the IPE. Electrodeposition was performed at a constant potential of −0.5 V for 120 s. Subsequently, the electrode was rinsed with ultrapure water to eliminate unanchored AuNPs and then dried it for subsequent use. Next, 5 μL of anti-Tau-411 at a concentration of 2 μg/mL was pipetted onto WE surface. The electrode was incubated at 4 °C overnight to ensure thorough antibody binding. The surface of the electrode was washed with PBS to remove any residues. Then, 5 μL of 10%wt BSA was added to block the unoccupied active sites on the surface of the modified electrode.

Finally, the prepared label-free electrochemical biosensor was stored at 4 °C for subsequent use.

### 2.2. Portable Electrochemical Detection Platform

**Figure 2:**
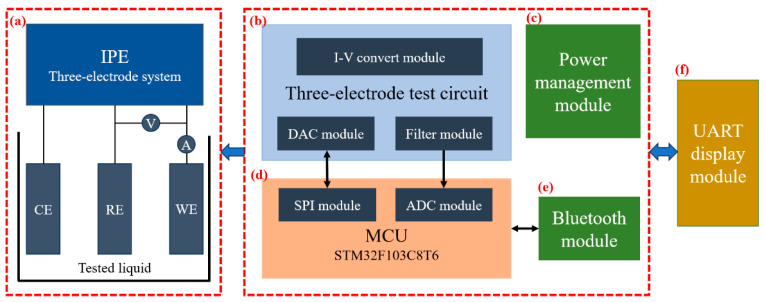
Schematic diagram of the electrochemical detection system module. (**a**) IPE for electrochemical detection system. (**b**) Three-electrode test circuit. (**c**) Power management module. (**d**) MCU and data processing module. (**e**) Bluetooth module. (**f**) UART display module.

As illustrated in Figure 2, the proposed system builds upon a previous experimental method by integrating six key modules into a fully functional mobile detection system [36]. (a) The three-electrode IPE, fabricated using inkjet-printing technology, was designed for electrochemical measurements. (b) The three-electrode detection circuit, serving as the core unit for signal processing in the system, mainly consisted of a reference voltage circuit, a constant potential circuit, a weak current detection circuit, and a filter circuit. (c) The power management module provided stable and adaptable multi-type voltage output for the system. (d) The MCU and data processing module employed the STM32F103C8T6 chip as the core processing unit, which undertook four key tasks: digital to analog converter (DAC) module configuration, signal acquisition, data processing, and instruction transmission. (e) The Bluetooth module utilized the low-power TB02 Bluetooth module to achieve wireless data transmission. (f) The UART display module enabled the real-time visual presentation of detection results and supported interface interaction functionality.

The three-electrode test circuit facilitated bidirectional electrochemical signal transmission, providing a stable operating environment for the test strip while simultaneously receiving its feedback current signal. The MCU communicated with the DAC via a built-in SPI interface to perform conversion, generating a high-precision control signal for the potentiostatic circuit. This circuit, constructed with a high-precision operational amplifier, produced a stable and accurate analog voltage signal, ensuring real-time control and exceptional performance in precision detection scenarios.

The MCU also acquired the voltage signal output from the three-electrode detection circuit and processed the raw data through filtering. The processed data was transmitted via a UART-connected Bluetooth module, meeting the requirements for real-time data transmission in field testing. Users could directly view detection results through a serial port display module, significantly improving operational convenience and user experience. Furthermore, the system incorporated a power management circuit built with multiple circuits, supporting various voltage levels to ensure stable operation of all modules in mobile working environments, thereby enhancing overall system reliability.

The circuit schematic diagrams (Figures S1 and S2) detail the hardware design and architecture of the main parts of the system. An operation video of this device is attached in the Supplementary File.

### 2.3. Electrochemical Immuno-Detection of Tau-441

To perform the immune determination, 10 μL of Tau-441 at various concentrations was dropped onto the IPE surface. The electrodes were incubated at 37 °C for 40 min to allow the specific formation of immunocomplexes. Subsequently, the electrodes were gently rinsed with PBS and deionized water to remove any non-specifically adsorbed proteins. The SWV measurements were then conducted in a 0.01 M PBS solution. The peak current decrease was recorded as the analytical signal for Tau-441 quantification.

## 3. Results and Discussion

### 3.1. Physical Characterization

**Figure 3:**
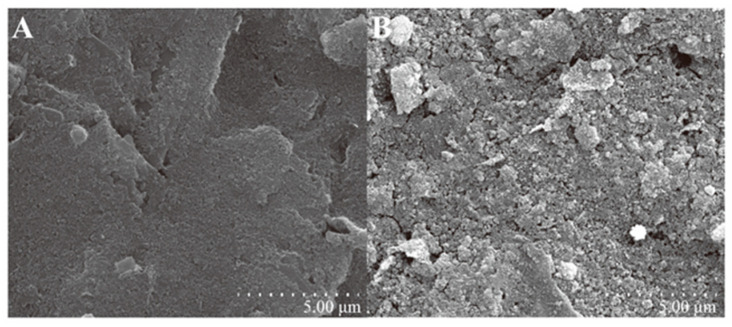
SEM images: (**A**) IPE; (**B**) Au NPs/IPE.

The morphology and structure of the nanocomposites were systematically characterized by scanning electron microscope (SEM). As shown in Figure 3A, the IPE was fabricated on a PET substrate using a graphene and PB composite ink. It exhibited an undulating, irregular, and high-surface-area morphology, consistent with the surface characteristics of typical polymer electrodes. In Figure 3B, the modified electrode surface was uniformly covered with a large number of densely distributed, uniformly sized, and irregularly arranged nanoparticles. These particles appeared brighter than the surrounding areas, indicating that Au NPs had been successfully loaded onto the electrode surface. This high-density distribution significantly provided abundant binding sites for antibody immobilization. As an excellent nanoscale-conductive medium, it also enhanced the electron transfer efficiency, significantly improving the response sensitivity and signal stability of the immunosensor.

**Figure 4:**
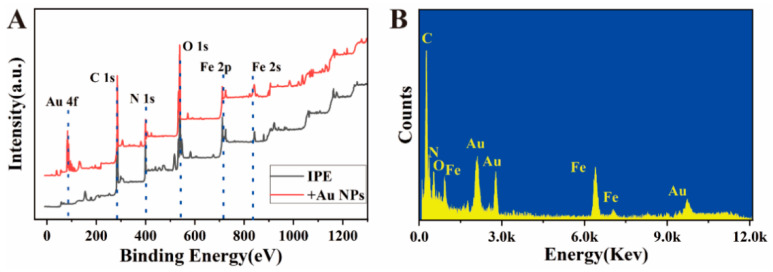
(**A**) XPS image and (**B**) EDS image.

Systematic characterization of the elemental composition through X-ray photoelectron spectroscopy (XPS) provides a more intuitive understanding of the successful modification of each functional layer and the interface composition. As shown in Figure 4A, the unmodified IPE (black curve) shows five characteristic peaks in the XPS spectrum, located at 285.1 eV (C 1s), 533.1 eV (O 1s), 399.1 eV (N 1s), 711.1 eV (Fe 2p), and 848.1 eV (Fe 2s). The C 1s and O 1s peaks mainly originate from the materials used in the electrode inkjet printing on the PET substrate; the N 1s and Fe 2p/2s signals are from the PB modification on the electrode surface, confirming its successful immobilization and providing electrochemically active centers for subsequent reactions. After the modification with Au NPs (red curve), in addition to the original characteristic peaks, a new pair of Au 4f characteristic peaks appears at a binding energy of approximately 89 eV, indicating that the Au NPs have been successfully and stably attached to the electrode surface. The XPS results are consistent with the SEM morphology observations, jointly demonstrating that the Au NPs form a uniform modification layer on the electrode surface, providing abundant active sites and a favorable biological interface support for subsequent antibody immobilization.

In Figure 4B, energy-dispersive spectroscopy (EDS) analysis further supplements the characterization from the perspective of elemental composition. It shows the distribution of elements such as C, N, and Fe in the material, and their content ratios are consistent with those of the electrode substrate and the materials used in the preparation process. Notably, the clear Au element signal in the EDS spectrum directly confirms that the Au NPs have been successfully and uniformly loaded onto the IPE surface. This result is mutually corroborated by the detection of the Au 4f characteristic peak in XPS, jointly constituting reliable evidence for the successful modification of Au NPs. Through this series of characterization methods, not only were the effective immobilization and distribution state of Au NPs on the electrode surface verified from multiple angles, but the physical structure, elemental composition, and chemical state of the modification layer were also systematically revealed. These structural advantages and interface characteristics jointly contribute to the excellent performance of the sensor in electrochemical detection.

### 3.2. Electrochemical Characterization of Immunosensors

**Figure 5:**
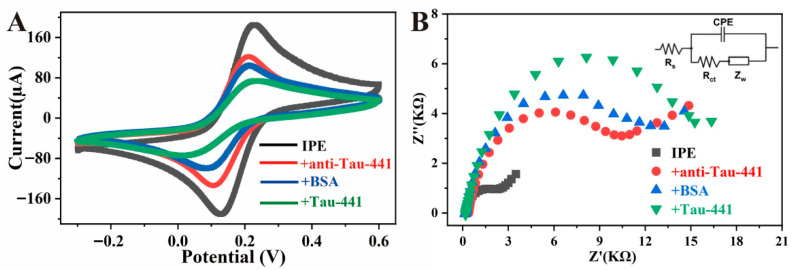
CV characterization (**A**) and EIS characterization (**B**) of different modification processes. (black) IPE, (red) IPE surface immobilized antibody, (blue) BSA blocking unbound active sites, (green) binding with antigen.

The stepwise modification process of the biosensor was systematically characterized by cyclic voltammetry (CV) and electrochemical impedance spectroscopy (EIS). As shown in the CV curve in Figure 5A, the PB working electrode modified with Au NPs (black curve) exhibited a pair of clear and symmetrical redox peaks at approximately 0.11 V and 0.22 V, corresponding to the redox reaction of ferricyanide/ferrocyanide in PB. Notably, the current response of these redox peaks was significantly higher than that without Au NPs modification, which was likely attributed to the excellent conductivity and large specific surface area of Au NPs, effectively promoting electron transfer and enhancing the electrode reaction kinetics. Subsequently, after the addition of antibodies to the electrode surface (red curve), the redox peak current decreased significantly, mainly due to the immobilization of antibody molecules on the Au NPs surface via Au-S bonds, partially blocking the electron transfer path. Then, after blocking the non-specific sites with BSA (blue curve), a further decrease in peak current was observed, indicating that BSA molecules successfully covered the remaining potential binding sites on the electrode, enhancing the specificity of the detection. After the immunorecognition reaction between the sensor and Tau-441 (green curve), the peak current decreased further. This change was mainly due to the formation of immune complexes by the specific binding of antigen and antibody, increasing the electron transfer resistance and significantly altering the electron transfer kinetics at the electrode surface. These regular electrochemical signal changes fully verified the successful construction of each modification layer of the sensor and its good recognition ability for the target antigen.

The Nyquist plot of EIS typically consists of a linear part at low frequencies (controlled by the diffusion process) and a semicircle at high frequencies (controlled by the electron transfer process). As shown in Figure 5B, the IPE modified with Au NPs (black curve) exhibited a smaller semicircle diameter, indicating a lower charge-transfer resistance (R_ct_), which was mainly attributed to the excellent conductivity and large specific surface area of Au NPs, effectively promoting interfacial electron transfer and significantly improving the electron transfer kinetics of the electrode. Subsequently, after the sequential immobilization of antibodies, BSA, and Tau-441, the semicircle diameter in the Nyquist plot gradually increased, indicating a continuous rise in R_ct_ values. This change reflected the stepwise construction of the biological recognition layer on the electrode surface. As Tau-441 binds specifically to the anti-Tau-441, the resulting macro-molecular complex acts as a physical and insulating barrier that impedes the access of charge carriers to the electrode surface, thereby increasing the charge-transfer resistance [36]. This result was highly consistent with the trend of the gradual decrease in peak current in CV, mutually verifying the changes in the interfacial properties of the sensor during the modification process and confirming the successful assembly of each functional layer.

### 3.3. Optimization of Sensor Detection Methods

**Figure 6:**
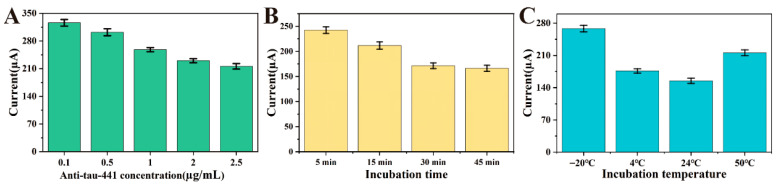
The influence of (**A**) antibody concentration, (**B**) incubation time and (**C**) incubation temperature on the detection performance of the sensor.

In this study, to optimize the performance of the immunosensing device, multiple key experimental parameters were optimized and detected by square wave voltammetry (SWV), including the amount of immobilized antibody, the incubation time and the incubation temperature of the antigen–antibody. Figure 6A shows the effect of antibody concentration on sensor performance. As the antibody concentration gradually increases, the SWV peak current response gradually decreases and stabilizes after reaching 2 μg/mL. This phenomenon indicates that the antibodies form a dense coverage layer on the electrode surface through stable coordination bonds with their thiol groups on the Au NPs surface, eventually reaching saturation. Moreover, the antibody molecules continuously occupy the effective binding sites on the Au NPs surface and gradually reach saturation coverage, leaving no vacant sites for further binding. Therefore, 2 μg/mL was selected as the optimal antibody concentration, providing a reliable basis for subsequent immunosensing experiments. This concentration ensures a sufficient number of capture probes while maintaining an appropriate molecular spacing, which avoids excessive steric hindrance that could occur at higher densities. Such balanced coverage facilitates the efficient accessibility of Tau-441 antigens to the antibody binding sites, thereby ensuring optimal sensitivity.

The specific binding of antigen–antibody requires a certain reaction time, so the peak current change can be used to screen the optimal incubation time to improve experimental efficiency. As shown in Figure 6B, when detecting 500 pg/mL Tau-441 at the optimal antibody concentration, it is observed that the electrochemical signal first gradually decreases and then stabilizes at around 30 min. As the incubation time continues to increase, the response current does not change significantly. This is determined by the kinetics of the antigen–antibody specific binding process. At the beginning of the reaction, the antibody concentration on the electrode surface is high, and the binding rate with the antigen is fast, resulting in a very obvious decrease in current. As time goes on, the available antibody binding sites on the electrode surface are gradually occupied by the antigen, and the local antigen concentration may also decrease due to binding. Therefore, the incubation time of the antibody–antigen was set to 30 min.

In addition, the effect of incubation temperature on detection performance was also studied Figure 6C. When detecting 500 pg/mL Tau-441 at the optimal antibody concentration, it is observed that the peak current of the electrochemical signal is the smallest at approximately 24 °C. At temperatures that are too low, the molecular thermal motion is insufficient, and the probability of antigen and antibody molecules colliding and binding is low, resulting in a slow reaction rate. At temperatures that are too high, proteins begin to denature and lose activity, and the three-dimensional structure of the antibody changes, possibly destroying the antigen-binding sites on it, leading to a decrease or even loss of specific binding ability. Therefore, the incubation temperature of the antigen–antibody was set to 24 °C and used as the standard experimental condition for subsequent experiments.

### 3.4. Performance Analysis of Immunosensors

**Figure 7:**
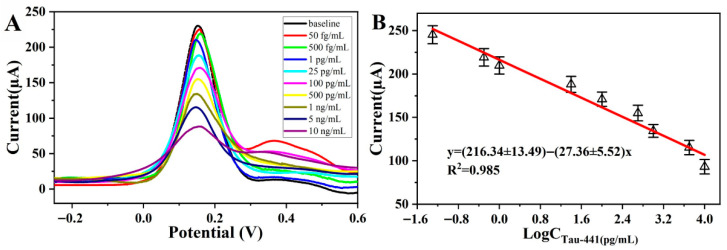
(**A**) Response currents of the electrochemical immunosensor under different concentrations of Tau-441 incubation: 0.05, 0.5, 1, 25, 100, 500, 1000, 5000, 10,000 pg/mL; (**B**) linear relationship between response current and logarithm of Tau-441 concentration.

To systematically evaluate the analytical detection performance of immunosensors, quantitative determination of different antigen concentrations was conducted under the optimal experimental conditions using SWV. As shown in Figure 7A, the SWV peak current response decreased regularly with the increase in antigen concentration. This change was attributed to the formation of immune complexes after the specific binding of antigens with immobilized antibodies, which hindered electron transfer and increased the interfacial electron transfer resistance. The sensor exhibited a favorable response relationship within the concentration range of 0.05 to 10,000 pg/mL, demonstrating an extremely wide linear detection range. The detection limit was far below the clinical warning concentration of AD-related biomarkers, a level sufficient to meet the sensitivity requirements for early diagnosis and dynamic monitoring. The wide linear response range also enhanced the efficiency and applicability in the detection of actual samples.

The linear relationship between the logarithm of antigen concentration and current response is shown in Figure 7B, and the regression equation is I(μA) = (216.34 ± 13.49) − (27.36 ± 5.52)logC_Tau-441_ (R^2^ = 0.985), with a detection limit of 16 fg/mL (S/N = 3). This value was determined using the formula LOD = 3σ/S, where σ is the standard deviation of the response for 10 blank samples and S is the slope of the calibration curve [37]. This result fully validates the extremely high detection sensitivity and reliability of the sensor. The low detection limit indicates that the sensor can effectively identify extremely low concentrations of target substances, providing a technical basis for the early diagnosis of AD. In addition, this biosensor is constructed based on electrochemical methods, with a relatively simple preparation process, good reproducibility, and low cost, making it suitable for large-scale batch detection in practical applications.

By comparing the method proposed in this study with the corresponding research in the published literature (Table 1), it can be clearly seen that the immunosensor prepared in this study not only shows a wider linear detection range but also has higher sensitivity. These advantages make it particularly suitable for practical screening and diagnosis scenarios for early AD. Moreover, the excellent performance of this sensor also provides a reliable tool and support for further exploration of the dynamic process of AD and quantitative research on related biomarkers.

### 3.5. Stability, Specificity and Repeatability

**Figure 8:**
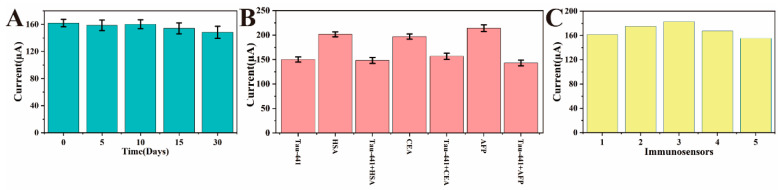
(**A**) Stability test of the immunosensor, stored in a refrigerator for 0, 5, 10, 15 and 30 days respectively; (**B**) specificity test of the immunosensor, 500 pg/mL Tau-441 was mixed with the same concentration of HSA, CEA and AFP respectively; (**C**) repeatability measurement of the immunosensor.

Stability is a key indicator for evaluating the performance of electrochemical immunosensors. In this study, the constructed immunosensor was stored at 4 °C for 30 days, and then tested for the same concentration of antigen. As shown in Figure 8A, the electrochemical signal did not show a significant decrease during this period, indicating that the sensor has good storage stability. This result suggests that the antibody can maintain its stability in a low-temperature environment, and the recognition site has not undergone significant denaturation or shedding. This stability can be attributed to (i) the strong coupling between the antibody and the nanostructured Au surface and (ii) effective blocking of nonspecific sites by BSA, which minimizes denaturation during storage. It also reflects the maintenance of the antigen–antibody binding ability and the high physical and chemical stability of the electrode interface modification layer. The sensor possesses excellent storage stability and batch-to-batch reproducibility. Given the low fabrication cost, these IPEs are designed for disposable use in point-of-care testing, effectively avoiding cross-contamination while ensuring reliable and precise diagnostic results.

To investigate whether the sensor has sufficient selectivity and anti-interference ability, common high-interference substances such as HSA, CEA, and AFP were selected as comparisons, and an equal mixture of antigens was also used for interference tests. The peak current bar chart shown in Figure 8B indicates that the peak current of the above interference substances has almost no change. This is due to the high specificity of antibody–antigen recognition and the effective surface blocking by BSA during the sensor preparation process. When the sensor was incubated with a mixed interference solution containing the target antigen, the change in peak current was very close to that of the control group containing only the target antigen, with no significant difference. The reason is that the affinity of specific binding is not affected, and the binding efficiency of the antibody binding site to the target antigen remains unchanged, indicating that the sensor can achieve specific detection of the target analyte. This is particularly important in actual clinical or complex biological matrices, ensuring the accuracy and reliability of the detection results.

In addition, the repeatability of the sensor was systematically evaluated. Five electrochemical sensors of the same batch were used to detect the same concentration of Tau-441. The results showed that the electrochemical responses were highly consistent and reproducible, indicating that the immunosensor has extremely high reliability in the construction process. This result also reflects the stability of the sensor preparation process, the consistency of antibody immobilization, and the precise control of the modification process, effectively reducing intra-batch differences. High repeatability is conducive to standardized measurement in actual detection environments, meeting the strict requirements for data accuracy and comparability in clinical diagnosis and long-term monitoring.

### 3.6. Determination of Tau-441 in Actual Samples

To evaluate the practical application value of the portable electrochemical immunosensor, the detection performance of Tau-441 in normal serum samples was analyzed in this study. Five serum samples were obtained from Guilin Medical University (Guilin, China), and the experiment was conducted in accordance with the guidelines of the institutional ethics committee. Different concentrations of Tau-441 were added to normal human serum samples by the spike-recovery method, and then the sample concentrations were measured using the portable immunosensor. The results are shown in Table 2. Recovery was evaluated via a standard spike-and-recovery approach. Known concentrations of Tau-441 (C_added_) were spiked into blank human serum, and the detected concentrations (C_found_) were obtained using the portable immunosensor. The recovery rate was calculated as follows:

Recovery (%) = C_found_/C_added_ × 100% (1)

As summarized in Table 2, recoveries ranged from 84.00% to 116.30% with RSD values below 10.12% (*n* = 5), demonstrating good matrix tolerance and analytical accuracy in actual serum samples.

## 4. Conclusions

This study constructed an electrochemical immunosensor based on IPEs functionalized with Au NPs for highly sensitive quantification of Tau-441. The Au NPs significantly enhanced interfacial electronic transport and increased antibody loading, forming a stable and efficient immunorecognition interface. Under optimized conditions, the sensor exhibited a wide linear range of 0.05–10,000 pg/mL and a detection limit of 16 fg/mL, along with excellent specificity, reproducibility, and operational stability. Furthermore, a portable, low-power electrochemical readout platform was also integrated to streamline the workflow from sample loading to result output. The sensor demonstrated strong correlation with reference methods in real-sample analyses, highlighting its potential for AD early screening. Furthermore, the detection platform developed by this research institute demonstrates significant economic and practical value. Compared to commercial electrochemical workstations with costs of several thousand dollars and expensive screen-printed electrodes, a single IPE has a material cost of approximately 0.2 US dollars, and the cost of the accompanying portable hardware is less than 20 US dollars. This provides a highly competitive solution for low-cost and large-scale early screening of AD. Although the batch-to-batch consistency of inkjet printing and its long-term antifouling stability in complex biological matrices still require systematic evaluation, subsequent efforts will focus on multicenter clinical validation and standardized quality control to facilitate practical application. Further optimization will be carried out in antifouling/anti-interference interface engineering, multimarker detection, and deep integration with portable platforms, ultimately establishing a scalable, low-cost technological pathway for early AD screening.

## Figures and Tables

**Table 1:** Comparison of performance parameters of different electrochemical methods for detecting Tau-441.

Biosensor	Linear Range (pg/mL)	LOD (pg/mL)	Detection Method	References
SPGE/VxPDA-MIP	0.005–0.15	0.002	EIS	[38]
SPCE/PBNCs/GO-MIP	50–100,000	0.46	SWV	[39]
ITO/PET	5000–100,000	4.3	DPV	[40]
Au/Cyst-PDICT	0.5–10,000	0.08	EIS	[41]
Au/Anti-Tau-441	0.05–10,000	0.016	SWV	This method

**Table 2:** Spiked detection of actual serum samples (*n* = 5).

Sample	Added (pg)	Founded (pg)	Recovery (%)	RSD (%)
1	1	0.84	84.00	10.12
2	10	11.63	116.30	8.66
3	100	105.49	105.49	4.17
4	500	504.34	100.87	1.92
5	1000	992.57	99.26	2.13

## Data Availability

The data of our study are available upon request.

## Supplementary Materials

The following supporting information can be downloaded at https://www.mdpi.com/article/10.3390/bios16020113/s1, Figure S1: Schematic diagram of the minimum system of MCU; Figure S2: Schematic diagram of weak current detection circuit; Video S1: Demonstration of portable device detection.
