# Supplementary material for: Inkjet-Printed Electrode Enable Portable Electrochemical Immunosensing of Tau-441 for Early Alzheimer’s Screening
Source: Biosensors (Basel). 2026 Feb 10;16(2):113. doi: 10.3390/bios16020113 (PMC12938625; doi:10.3390/bios16020113)
Supplement: Supplementary file 1 [file biosensors-16-00113-s001.zip › biosensors-4090995-Supplementary.pdf]

biosensors

MDPI

Supplementary Materials

1. Inkjet-Printed Electrode Enable Portable Electrochemical Immunosensing of Tau-441 for Early Alzheimer's Screening

Binglun Li [1,2,3], Chenghao Liu [1,2,3], Chenlu Gu [4], Shanshan Wei [2,3,5], Shiyong Li [2,3,4], Ziang Liu [1,2,3], Dongdong Zhao [1,2,3], Qunfeng Tang [1,2,3], Yun Chen [6,*] and Zhencheng Chen [1,2,3,5,*]

[1] School of Life and Environmental Sciences, Guilin University of Electronic Technology, Guilin 541004, China; lbl@guet.edu.cn (B.L.); lchjky@163.com (C.L.); ziiangliu@163.com (Z.L.); dongzhaochd@163.com (D.Z.); tangqunfeng1771@foxmail.com (Q.T.)
[2] Guangxi Colleges and Universities Key Laboratory of Biomedical Sensors and Intelligent Instrument, Guilin 541004, China; wei.shanshan@outlook.com (S.W.); lishiyong@guet.edu.cn (S.L.)
[3] Guangxi Human Physiological Information Non-Invasive Detection Engineering Technology Research Center, Guilin 541004, China
[4] School of Electronic Engineering and Automation, Guilin University of Electronic Technology, Guilin 541004, China; chenlugu7@gmail.com
[5] School of Artificial Intelligence Medicine, Guilin Medical University, Guilin 541199, China
[6] School of Physics and Technology, Guangxi Normal University, Guilin 541004, China
* Correspondence: yun_chen2022@163.com (Y.C.); chenzhcheng@guet.edu.cn (Z.C.)

S1. Reagents

All chemicals used in this study were of analytical grade. Tau-441 was purchased from BPS Bioscience. Anti-Tau-441 was obtained from Biotechnology Co., Ltd. BSA was purchased from Sigma-Aldrich (Shanghai) Trading Co., Ltd. Human serum albumin (HSA), carcinoembryonic antigen (CEA), alpha-fetoprotein (AFP), graphene, Prussian blue (PB), and $HAuCl_4$ were obtained from Shanghai Sangon Biotechnology Co., Ltd. Sulfuric acid (H_2SO_4) and PBS were purchased from Sinopharm Chemical Reagent Co., Ltd.

S2. Apparatus

All electrochemical tests, including electrochemical impedance spectroscopy (EIS), cyclic voltammetry (CV), and square wave voltammetry (SWV), were conducted using an electrochemical workstation (Versa STAT 3, Princeton Applied Research, USA). Energy dispersive spectroscopy (EDS) characterization was performed using a scanning electron microscope (SEM, Mira, TESCAN, Czech Republic). X-ray photoelectron spectroscopy (XPS) characterization was carried out using a K-Alpha from Thermo Fisher Scientific (China) Co., Ltd. Antibody incubation in the experiments was conducted using a constant temperature incubator (BSD-100, FEI Company, USA). IPE was accomplished using an HP DeskJet 1212 Printer. Ultra-pure water for the experiments was produced by a Kertone-MINI8-Y ultra-pure water purification system (Hunan Kerton Water Technology Co., Ltd) with a resistivity of 18.2 MΩ • cm.

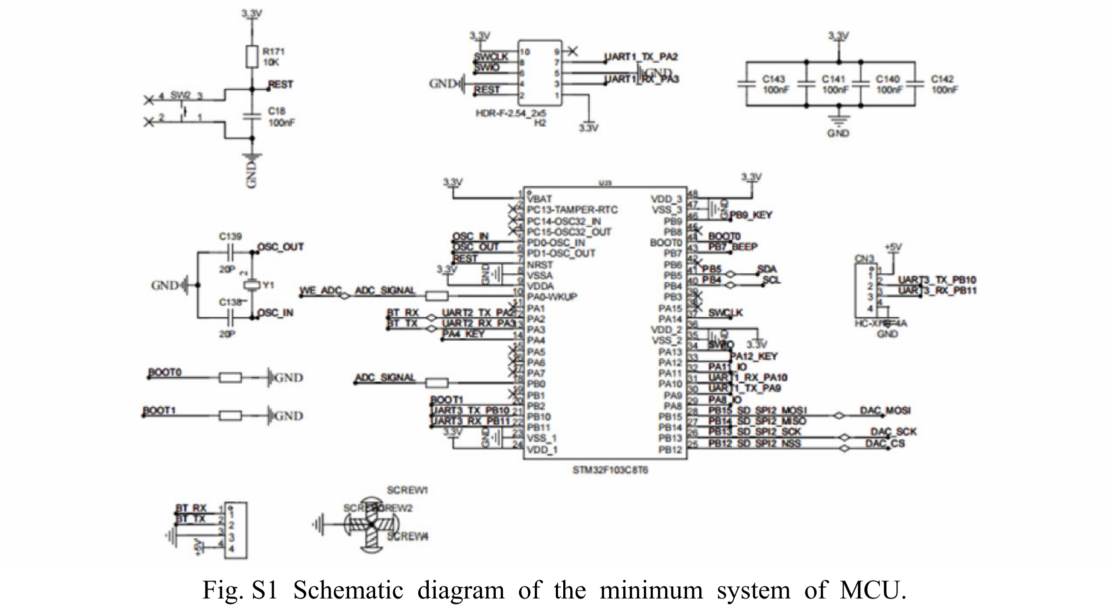

Fig. S1 Schematic diagram of the minimum system of MCU.

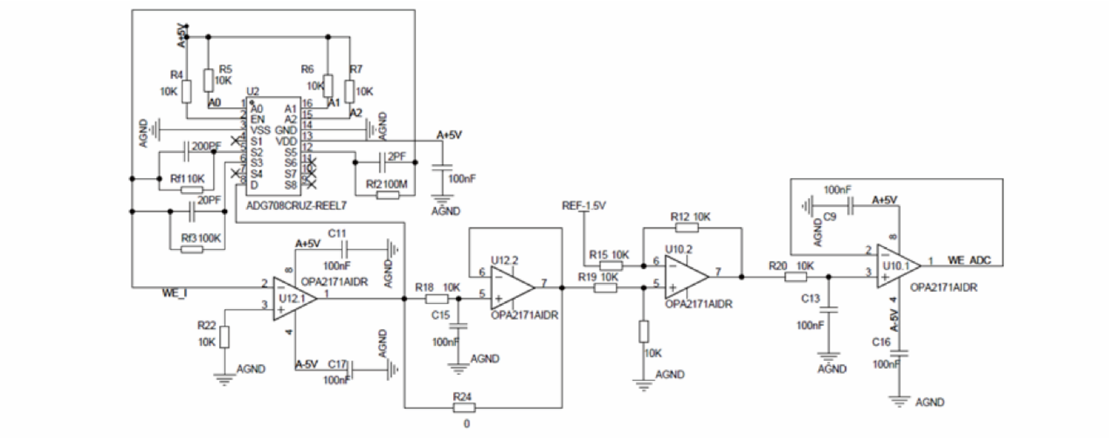

Fig. S2 Schematic diagram of weak current detection circuit.
